# The impact of substance use disorder on the mental health among COVID-19 patients: A protocol for systematic review and meta-analysis

**DOI:** 10.1097/MD.0000000000023203

**Published:** 2020-11-13

**Authors:** Yun Jin Kim, Linchao Qian, Muhammad Shahzad Aslam

**Affiliations:** School of Traditional Chinese Medicine, Xiamen University Malaysia, Jalan Sunsuria, Bandar Sunsuria, Sepang, Selangor, Malaysia.

**Keywords:** adolescents, COVID-19, mental health, substance use disorder

## Abstract

Substance use disorder (SUD) is associated with a high risk of physical and mental illness such as anxiety, depression, personality disorders, eating disorders, and abnormal mood changes. During the pandemic, SUD, a significant problem related to Coronavirus disease 2019 (COVID-19), is affecting adolescents. The recent available literature also emphasizes understanding the relationship between mental illness and SUD. Hence, it is essential to evaluate the scientific approach and examine the presented findings of articles published on SUD during the COVID-19 pandemic. A systematic review will be conducted using PubMed, PubMed Central, and Scopus bibliographic databases. The grey literature on the impact of SUD on mental health during the COVID-19 pandemic among adolescents will be identified using scholar google. The dependability and credibility of the findings will be examined using the ConQual approach. The methodologies of the included studies will be compared using ROBIS (risk of bias in systematic reviews tool), a measurement tool to assess systematic reviews (AMSTAR), and the JBI critical appraisal tool. The systematic review will be carried out on published articles, so it is exempt from ethics approval. The Center for Open Science (OSF) will be used as a data repository during the preparation of the protocol and completion of the systematic review. The research findings will be published in a related peer-reviewed journal.

## 1 Introduction

The risk of morbidity and mortality of mental disorders increases with substance misuse.^[1–3]^ Positive comorbidity has also been observed between psychiatric and substance use among men (48%) and women (47%), having an antisocial personality disorder (25.1%), and major depression (15.8%), respectively.^[4]^ A recent national-wide cross-sectional study identified the prevalence of substance-induced anxiety (weighted Prevalence 5.0%, 4.2–5.8) in China.^[5]^ Several systematic reviews have also confirmed the prevalence of comorbid mental health disorders in people having substance abuse.^[6]^

The advent and spread of the 2019 novel coronavirus (2019-nCoV) caused by severe acute respiratory syndrome 2 (SARS-CoV-2) pose major public health issues and a global pandemic.^[7]^ According to the worldometer, there have been 599,438 deaths (n = 599,438) caused by Coronavirus disease 2019 (COVID-19) and 14,194,726 infected (n = 14,194,726) as of the 18th July 2020.^[8]^ The World Health Organization has recommended reducing watching, reading, or listening to news that makes anyone anxious and distressed.^[9]^

Several commentaries, correspondence, communications, editorials, and letters published in high impact journals have made prominent the effect of COVID-19 on mental health.^[10]^ There will be an increase in the desire for substance abuse, such as smoking, because of social isolation and psychic distress.^[11]^ The rise in stress could lead to other substance abuse, such as alcohol misuse caused because of the improper functioning of the cortisol response and emotional regulation.^[12,13]^ In addition to smoking and alcohol, the patients suffering from addiction and dependence, such as opioid use disorder, may face a challenge for treatment.^[14–16]^ A national-wide case-control study in Korea has also depicted several comorbidities associated with COVID-19, including substance use (ORR, 1.321–1.381).^[17]^ The list of physical and mental issues related to COVID-19 consists of anxiety, stigma, and stress, which could lead to domestic violence,^[18]^ boredom,^[19]^ physical inactivity,^[20]^ and time management issues.^[9,21]^

### 1.1 Objectives

The objectives of this study are as follows:

1. To identify the substance use among adolescents during the COVID-19 pandemic.
2. To examine the quality assessment of original articles.
3. To compare the different quality assessment methods of the included studies.
4. To give future direction on how to improve the credibility of the findings on the mental issues among adolescents during a global pandemic.

### 1.2 Inclusion criteria

Original research articles published in the English language in which substance use disorder and COVID-19 were considered as the primary measured variables. Review articles, commentary, correspondence, communication, editorials, opinions, and letters will be excluded.

### 1.3 Participants

This systematic review will target studies in which groups or subgroups of the participants are all 3 kinds of adolescents, such as early adolescence, middle adolescence, and late adolescence.

### 1.4 Context

No time limit for setting the study or timeframe will be set for this systematic review. The psychological assessment questionnaires (ie, generalized anxiety disorder), mental and behavioral disorders and their methods to evaluate their characteristics will be included in the context of our systematic review.

### 1.5 Type of studies

Articles that have been published as open access and written in English will be selected under this systematic review, and there will be no limitation on the date of acceptance or publication. The researchers will consider only articles that have been published or are in the press.

### 1.6 Information source

An electronic search will be performed through PubMed,^[22]^ PubMed Central,^[23]^ and Scopus database,^[24]^ whereas gray literature will be identified using Scholar Google.^[25]^ ProQuest will be searched for dissertations.^[26]^

### 1.7 Search strategy

Our initial search syntax for PubMed will be:

1. ((((“substance-related disorders”[MeSH Terms] OR (“substance related”[All Fields] AND “disorders”[All Fields])) OR “substance related disorders”[All Fields]) OR (“substance”[All Fields] AND “disorder”[All Fields])) OR “substance use disorder”[All Fields]) AND (((((((“covid 19”[All Fields] OR “covid 2019”[All Fields]) OR “severe acute respiratory syndrome coronavirus 2”[Supplementary Concept]) OR “severe acute respiratory syndrome coronavirus 2”[All Fields]) OR “2019 ncov”[All Fields]) OR “sars cov 2”[All Fields]) OR “2019ncov”[All Fields]) OR ((“wuhan”[All Fields] AND (“coronavirus”[MeSH Terms] OR “coronavirus”[All Fields])) AND (2019/12/1:2019/12/31[Date - Publication] OR 2020/1/1:2020/12/31[Date - Publication])))
2. ((((“substance-related disorders”[MeSH Terms] OR (“substance related”[All Fields] AND “disorders”[All Fields])) OR “substance related disorders”[All Fields]) OR (“drug”[All Fields] AND “addiction”[All Fields])) OR “drug addiction”[All Fields]) AND (((((((“covid 19”[All Fields] OR “covid 2019”[All Fields]) OR “severe acute respiratory syndrome coronavirus 2”[Supplementary Concept]) OR “severe acute respiratory syndrome coronavirus 2”[All Fields]) OR “2019 ncov”[All Fields]) OR “sars cov 2”[All Fields]) OR “2019ncov”[All Fields]) OR ((“wuhan”[All Fields] AND (“coronavirus”[MeSH Terms] OR “coronavirus”[All Fields])) AND (2019/12/1:2019/12/31[Date - Publication] OR 2020/1/1:2020/12/31[Date - Publication])))
3. ((((“illicit drugs”[MeSH Terms] OR (“illicit”[All Fields] AND “drugs”[All Fields])) OR “illicit drugs”[All Fields]) OR (“illicit”[All Fields] AND “drug”[All Fields])) OR “illicit drug”[All Fields]) AND (((((((“covid 19”[All Fields] OR “covid 2019”[All Fields]) OR “severe acute respiratory syndrome coronavirus 2”[Supplementary Concept]) OR “severe acute respiratory syndrome coronavirus 2”[All Fields]) OR “2019 ncov”[All Fields]) OR “sars cov 2”[All Fields]) OR “2019ncov”[All Fields]) OR ((“wuhan”[All Fields] AND (“coronavirus”[MeSH Terms] OR “coronavirus”[All Fields])) AND (2019/12/1:2019/12/31[Date - Publication] OR 2020/1/1:2020/12/31[Date - Publication])))
4. ((((“substance-related disorders”[MeSH Terms] OR (“substance related”[All Fields] AND “disorders”[All Fields])) OR “substance related disorders”[All Fields]) OR (“substance”[All Fields] AND “abuse”[All Fields])) OR “substance abuse”[All Fields]) AND (((((((“covid 19”[All Fields] OR “covid 2019”[All Fields]) OR “severe acute respiratory syndrome coronavirus 2”[Supplementary Concept]) OR “severe acute respiratory syndrome coronavirus 2”[All Fields]) OR “2019 ncov”[All Fields]) OR “sars cov 2”[All Fields]) OR “2019ncov”[All Fields]) OR ((“wuhan”[All Fields] AND (“coronavirus”[MeSH Terms] OR “coronavirus”[All Fields])) AND (2019/12/1:2019/12/31[Date - Publication] OR 2020/1/1:2020/12/31[Date - Publication])))
5. ((“drug misuse”[MeSH Terms] OR (“drug”[All Fields] AND “misuse”[All Fields])) OR “drug misuse”[All Fields]) AND (((((((“covid 19”[All Fields] OR “covid 2019”[All Fields]) OR “severe acute respiratory syndrome coronavirus 2”[Supplementary Concept]) OR “severe acute respiratory syndrome coronavirus 2”[All Fields]) OR “2019 ncov”[All Fields]) OR “sars cov 2”[All Fields]) OR “2019ncov”[All Fields]) OR ((“wuhan”[All Fields] AND (“coronavirus”[MeSH Terms] OR “coronavirus”[All Fields])) AND (2019/12/1:2019/12/31[Date - Publication] OR 2020/1/1:2020/12/31[Date - Publication])))
6. (“deviant”[All Fields] OR “deviants”[All Fields] drug use) AND (“COVID-19”[All Fields] OR “COVID-2019”[All Fields] OR “severe acute respiratory syndrome coronavirus 2”[Supplementary Concept] OR “severe acute respiratory syndrome coronavirus 2”[All Fields] OR “2019-nCoV”[All Fields] OR “SARS-CoV-2”[All Fields] OR “2019nCoV”[All Fields] OR ((“Wuhan”[All Fields] AND (“coronavirus”[MeSH Terms] OR “coronavirus”[All Fields])) AND (2019/12[PDAT] OR 2020[PDAT])))
7. ((((“substance-related disorders”[MeSH Terms] OR (“substance related”[All Fields] AND “disorders”[All Fields])) OR “substance related disorders”[All Fields]) OR (“drug”[All Fields] AND “dependence”[All Fields])) OR “drug dependence”[All Fields]) AND (((((((“covid 19”[All Fields] OR “covid 2019”[All Fields]) OR “severe acute respiratory syndrome coronavirus 2”[Supplementary Concept]) OR “severe acute respiratory syndrome coronavirus 2”[All Fields]) OR “2019 ncov”[All Fields]) OR “sars cov 2”[All Fields]) OR “2019ncov”[All Fields]) OR ((“wuhan”[All Fields] AND (“coronavirus”[MeSH Terms] OR “coronavirus”[All Fields])) AND (2019/12/1:2019/12/31[Date - Publication] OR 2020/1/1:2020/12/31[Date - Publication])))

### 1.8 Assessment of methodological quality

#### 1.8.1 ROBIS

The systematic review tool (ROBIS) is one of the newly developed tools to assess the risk of bias in the systematic review which consists of three main parameters (assess the relevance, identify concern and judge the risk of bias).^[27]^

#### 1.8.2 AMSTAR

A measurement tool for the “assessment of multiple systematic reviews,” AMSTAR is used to assess the quality of the manuscript using an improved quality assessment questionnaire that consists of 11 items.^[28]^

### 1.9 JBI critical appraisal tool

The Joanna Briggs Insititute has constructed 10-item critical appraisal tools to evaluate the philosophical perspective, research questions or objectives, methods used to collect data, data analysis, and interpretation of the findings.^[29]^

### 1.10 Data synthesis

Preferred Reporting Items for Systematic Reviews and Meta-Analyses (PRISMA) guidelines will be used to prepare the final manuscript.^[30]^ Qualitative Data Analysis Software (QDA Miner) will be used to aid the data extraction ^[31]^

### 1.11 Examining confidence in the findings

The final synthesized findings will be marked according to the ConQual approach for establishing confidence in the output of the qualitative research synthesis and presented in a summary of findings.^[32]^ Moreover, the initial screening will be performed with the help of 2 independent reviewers.

### 1.12 Expected outcome

The reviewer will able to identify the impact of anxiety, depression, and attitude among adolescents against the COVID-19 pandemic from the baseline to the last available follow-up. Measures of the effects relative to the risks, odds ratios, and the risk difference of anxiety, depression, and attitude before and after COVID-19 will be observed.

## Acknowledgments

The authors gratefully acknowledge Xiamen University Malaysia for their support and guidance. Special thanks to Aishah Rose Marie, for her perseverance in proofreading the manuscript.

## Author contributions

**Conceptualization:** Yun Jin Kim, Linchao Qian, Muhammad Shahzad Aslam.

**Data curation:** Linchao Qian.

**Funding acquisition:** Yun Jin Kim.

**Methodology:** Linchao Qian.

**Supervision:** Linchao Qian, Muhammad Shahzad Aslam.

**Writing – original draft:** Yun Jin Kim, Muhammad Shahzad Aslam.

**Writing – review & editing:** Yun Jin Kim, Muhammad Shahzad Aslam.
